# Use and cost comparison of clobazam to other antiepileptic drugs for treatment of Lennox-Gastaut syndrome

**DOI:** 10.1080/20016689.2017.1318691

**Published:** 2017-05-19

**Authors:** Clément François, John M. Stern, Augustina Ogbonnaya, Tasneem Lokhandwala, Pamela Landsman-Blumberg, Amy Duhig, Vivienne Shen, Robin Tan

**Affiliations:** ^a^ Health Economics and Outcomes Research, Lundbeck, LLC, Deerfield, IL, USA; ^b^ Department of Neurology, University of California, Los Angeles, Los Angeles, CA, USA; ^c^ Scientific Consulting, Xcenda, LLC, Palm Harbor, FL, USA

**Keywords:** Lennox-Gastaut syndrome, seizure, antiepileptic drugs, clobazam, seizure-related costs

## Abstract

**Background**: Lennox-Gastaut syndrome (LGS) is a severe form of childhood-onset epilepsy associated with serious injuries due to frequent and severe seizures. Of the antiepileptic drugs (AEDs) approved for LGS, clobazam is a more recent market entrant, having been approved in October 2011. Recent AED budget impact and cost-effectiveness analyses for LGS suggest that adding clobazam to a health plan formulary may result in decreased medical costs; however, research on clinical and economic outcomes and treatment patterns with these AED treatments in LGS is limited.

**Objectives**: To compare the baseline characteristics and treatment patterns of new initiators of clobazam and other AEDs among LGS patients and compare healthcare utilization and costs before and after clobazam initiation among LGS patients.

**Methods**: A retrospective study of probable LGS patients was conducted using the MarketScan® Commercial, Medicare Supplemental, and Medicaid databases (10/1/2010-3/31/2014).

**Results**: In the Commercial/Medicare Supplemental population, clobazam users were younger, had fewer comorbidities, and more prior AED use than non-clobazam users. In the 12 months pre-treatment initiation, clobazam users had significantly more seizure-related inpatient stays and outpatient visits and higher total seizure-related (*P* < 0.001) and all-cause (*P* < 0.001) costs than non-clobazam users. Among clobazam users, when compared to the 12 months pre-clobazam initiation, seizure-related medical utilization and costs were lower in the 12 months post-clobazam initiation (*P* = 0.004). Total all-cause (*P* < 0.001) and seizure-related (*P* = 0.029) costs increased post-clobazam initiation mainly due to the increase in outpatient pharmacy costs. Similar results were observed in the Medicaid population.

**Conclusions**: Baseline results suggest a prescribing preference for clobazam in severe LGS patients. Clobazam users had a reduction in seizure-related medical utilization and costs after clobazam initiation. The improvement in medical costs mostly offset the higher prescription costs following clobazam initiation.

## Background

Lennox-Gastaut syndrome (LGS) is a severe type of epilepsy with onset in childhood and characterized by intellectual disability, specific electroencephalographic abnormalities, and frequent generalized-onset or sometimes focal-onset seizures [1]. LGS typically develops before eight years of age, with occurrence rates peaking between three and five years of age; however, late cases in early adulthood have also been reported [1]. Estimated to account for 1–10% of all childhood epilepsies, LGS has a mortality rate between 4% and 7% in patients younger than 11 years of age [2–4].

LGS can have a major physical impact due to frequent and severe seizures that increase the likelihood of fall-related injuries. Seizures during the childhood development stage can halt cognitive and social development and lead to behavioral impairment [1,5]. Cognitive impairment is seen in 75–95% of patients five years after condition onset, and 90% will eventually become intellectually disabled [5,6]. Additionally, LGS has a significant impact on the health-related quality of life of not only the patient but also their caregiver [7].

Six antiepileptic drugs (AEDs) have been approved to date by the United States Food and Drug Administration (FDA) for the treatment of LGS: clobazam; clonazepam; felbamate; lamotrigine; topiramate; and rufinamide. Valproate and clorazepate, which are not approved by the FDA for LGS treatment, are routinely used in these patients [8,9]. Of the approved drugs, clobazam is a more recent market entrant, having been approved in October 2011 for use as an adjunctive treatment for seizures associated with LGS in adults and children two years of age and older.

Clobazam has demonstrated both short- and long-term efficacy in children, adolescents, and adults, and is well tolerated across all ages [10]. Recent AED budget impact and cost-effectiveness analyses for LGS suggest that adding clobazam to a health plan formulary may have a positive overall budget impact through decreased medical costs, as clobazam has been proven efficacious in the treatment of drop seizures, which are a major cause of morbidity and healthcare utilization among these patients [11,12]. Clobazam has specifically been shown to be more effective and less costly than rufinamide over a two-year period [11].

Currently, there is limited research on clinical and economic outcomes and a lack of information on treatment patterns with the aforementioned AED treatments in LGS. Therefore, this study had two objectives: (1) to describe the baseline characteristics and treatment patterns of LGS patients treated with clobazam compared to other AED treatments; and (2) to compare healthcare resource utilization and costs before and after treatment among clobazam users.

## Methods

### Study design

This retrospective longitudinal cohort study used administrative claims data with dates of service from 1 October 2010 through 31 March 2014. The date treatment of interest initiated was set to be the index date. The study enrollment period was between 1 October 2011 and 30 September 2013, allowing a 12-month pre-treatment initiation period (i.e., pre-index) and a minimum six-month post-treatment initiation (i.e., post-index) period.

### Data source

This study was conducted using three Truven Health MarketScan® claims databases [12]: the Commercial Claims and Encounters Database (Commercial); the Medicare Supplemental and Coordination of Benefits Database (Medicare Supplemental); and the Medicaid Multi-state Database (Medicaid). The Commercial database consists of employer- and health plan-sourced data containing medical and drug claims for over 40 million individuals annually. The Medicare Supplemental database contains the medical and prescription claims of Medicare-eligible persons with supplemental insurance offered by their former employers. There are approximately 4.3 million enrollees annually included in the database. For the purposes of these analyses, the Commercial and Medicare Supplemental databases were combined and a single unique identifier allows for patients to be followed as they move from a Commercial payer to Medicare. The Medicaid database contains the medical and prescription drug experience of Medicaid enrollees in both fee-for-service and managed-care plans pooled from 10 to 13 states annually.

### Patient identification

Criteria used to identify patients with LGS were based on a previously published algorithm [13] and clinical input (John M. Stern and Vivienne Shen), and are as follows: (1) ≥2 medical claims with a diagnosis of generalized convulsive (International Classification of Diseases, Ninth Revision, Clinical Modification [ICD-9-CM: 345.1x]) or non-convulsive epilepsy (ICD-9-CM: 345.0x) that are ≥30 days apart, or ≥1 medical claim with a diagnosis of generalized convulsive epilepsy (ICD-9-CM: 345.1x) and ≥1 medical claim with a diagnosis of non-convulsive epilepsy (ICD-9-CM: 345.0x) that are ≥30 days apart; (2) ≥1 of the epilepsy diagnosis codes had to be in the primary position; (3) ≥1 medical claim with a diagnosis for developmental disorder or cognitive impairment (ICD-9-CM: 299.80, 299.81, 299.90, 299.91, 294.8x, 294.9x, 315.39, 315.4x, 315.5x, 315.8x, 315.9x, 316.xx, 317.xx, 318.0x, 318.1x, 318.2x, 319.xx, 348.3x, 348.89, 780.97, and 783.4x) in any position during the enrollment window.

Based on the AED treatment initiated during the enrollment window and after the first evidence of LGS diagnosis, patients were further classified into one of two mutually exclusive treatment groups: clobazam or non-clobazam. Patients in the clobazam cohort initiated treatment with clobazam, and may have received prior treatment with another AED. The date of the first clobazam prescription fill was deemed the index date. Patients in the non-clobazam cohort initiated treatment with another AED (clonazepam, felbamate, lamotrigine, rufinamide, topiramate, valproate, or clorazepate). To qualify as the index prescription, the patient had to be naïve to that particular AED (i.e., have no other prescription claims for that AED in the prior 12 months); otherwise, the subsequent AED prescription was evaluated similarly. Lastly, patients must have continuous enrollment in medical and pharmacy benefits for ≥12 months prior to and ≥6 months after the index date. For the pre- and post-clobazam healthcare resource utilization and cost comparison, clobazam patients had to have ≥12 months of post-index continuous enrollment. Patients were excluded if they did not have a qualifying AED prescription during the enrollment window or if they were dually eligible for both Medicare and Medicaid, as prescription claims were not available for these patients.

### Study measures

#### Demographic and clinical characteristics

Demographic characteristics, including age, gender, race (Medicaid only), geographic region (Commercial/Medicare Supplemental only), urban/rural, plan type (Commercial/Medicare Supplemental only), payer type (Commercial/Medicare Supplemental only), capitation, reason for eligibility (Medicaid only), length of follow-up, and year of index date, were measured on index for each patient. Pre-index clinical characteristics included use of medication classes (other seizure medications, anxiolytics, antipsychotics, and hypnotics), number of unique medication classes used, number of AED drugs of interest, and comorbidities.

#### Healthcare resource utilization and costs

Healthcare resource utilization and costs were conducted from the perspective of a private or governmental insurance plan in the United States. Per-patient all-cause and seizure-related healthcare resource utilization and their associated costs were reported by treatment setting (e.g., inpatient, emergency room [ER], physician office, laboratory, radiology, other outpatient, and pharmacy) during the pre-index period and also for the clobazam cohort during the post-index period. All-cause resource utilization included all medical and pharmacy services for any reason during the time period of interest. Medical resource utilization was deemed to be seizure-related if the primary ICD-9-CM diagnosis code on a claim was 345.0x–345.9x, and seizure-related pharmacy services included all prescriptions for the AEDs of interest and other seizure-related medications (Table 1). All-cause healthcare costs were costs associated with all utilizations, while seizure-related healthcare costs were costs for all seizure-related utilizations. Costs reflected all payments made to providers of care from both the plan (plan and coordination of benefits) and the patient (copayment, coinsurance, deductible). All costs were adjusted to 2014 United States dollars (USD) using the medical care component of the Bureau of Labor Statistics’ Consumer Price Index.

**Table 1.** Antiseizure medications.

Medication/medication class	Generic name (brand)
AMPA receptor antagonist	Perampanel (Fycompa)
SVP2a binder	Levetiracetam (Keppra, Keppra XL)
Carbonic anhydrase inhibitor	Acetazolamide (Diamox)
Sodium channel inhibitor	Carbamazepine (Tegretol), Eslicarbazepine (Aptiom), Oxcarbazepine (Trileptal), Rufinamide (Banzel)
GABA analogues	Gabapentin (Neurontin), Pregabalin (Lyrica), Progabide (Gabrene), Vigabatrin (Sabril)
GABA reuptake inhibitors	Tiagabine (Gabitril)
K-channel opener	Ezogabine/Retigabine (Potiga)
NMDA receptor blockers	Felbamate (Felbatol), Sodium Channel Modulators, Lacosamide (Vimpat), Lamotrigine (Lamictal), Phenytoin (Dilantin)
T-type calcium channel	Ethosuximide (Zarontin), Methsuximide (Celontin)
Sulfamate-substituted monosaccharides	Topiramate (Topamax, Topamax ER, Qudexy XR)
Sodium channel	Zonisamide (Zonegran)
Valproic acid	Divalproex Sodium (Depakote), Valproic Acid (Depakene)
Barbiturates	Phenobarbital, Primidone (Mysoline)
Benzodiazepines	Clobazam (Onfi), Clonazepam (Klonopin, Epitril, Rivotril), Clorazepate Dipotassium (Tranxene), Diazepam (Valium, Diastat), Lorazepam (Ativan)

Notes: GABA – gamma aminobutyric acid; NMDA – N-methyl-D-aspartate; SVP2a – synaptic vesicle protein 2A.

#### Treatment patterns

Treatment patterns were assessed for both study cohorts during the post-index period and included the number of index AED prescription claims, total days on index drug and all AEDs, changes to the index treatment (i.e., augmentation, switching, discontinuation), and discontinuation from all AEDs. Augmentation was defined as a prescription fill for an alternative AED treatment (one of the seven non-index AEDs) while continuing to fill prescriptions for the index AED. Switching was defined as a prescription fill for a new AED that was different from the index agent, with no further prescription fills for the index AED. Discontinuation from index treatment and from all AEDs was defined as a >30-day period without evidence of having the index AED and or any AEDs on hand respectively.

### Statistical analyses

Separate analyses were conducted for the combined Commercial and Medicare Supplemental and Medicaid populations.

#### Clobazam vs non-clobazam cohorts

Descriptive statistics (percentages, means, medians, standard deviations) were used to describe the baseline characteristics of the clobazam and non-clobazam cohorts. Statistical comparison of baseline measures was conducted using chi-squared tests for categorical measures and Student’s *t*-tests or Mann-Whitney tests for continuous measures, as appropriate to the underlying distribution. To create comparability of the baseline demographic and clinical characteristics of clobazam and non-clobazam users for treatment pattern comparisons, standardized mortality ratio weighting, a propensity score (PS) technique, was employed in order to ameliorate selection bias prior to comparison of outcomes [14]. PS for each patient was estimated using a logistic regression model that included patients’ baseline characteristics as the independent variables and treatment as the dependent variable. Weights were computed as follows: 1 (PS/PS) for clobazam users and PS/(1 − PS) for the non-clobazam users.

As baseline characteristics between the clobazam and non-clobazam cohorts remained unbalanced, for treatment patterns during the post-index period, descriptive statistics were presented for the clobazam and non-clobazam cohorts, and no statistical comparison was performed.

#### Healthcare resource utilization and costs pre- and post-clobazam initiation

Among the clobazam cohort, healthcare resource utilization, and costs pre- and post-treatment initiation were compared. Statistical differences in categorical measures were analyzed using McNemar’s tests, count measures using paired *t*-tests, and costs using Wilcoxon signed-rank tests.

## Results

### Baseline demographic characteristics

**Figure 1.**
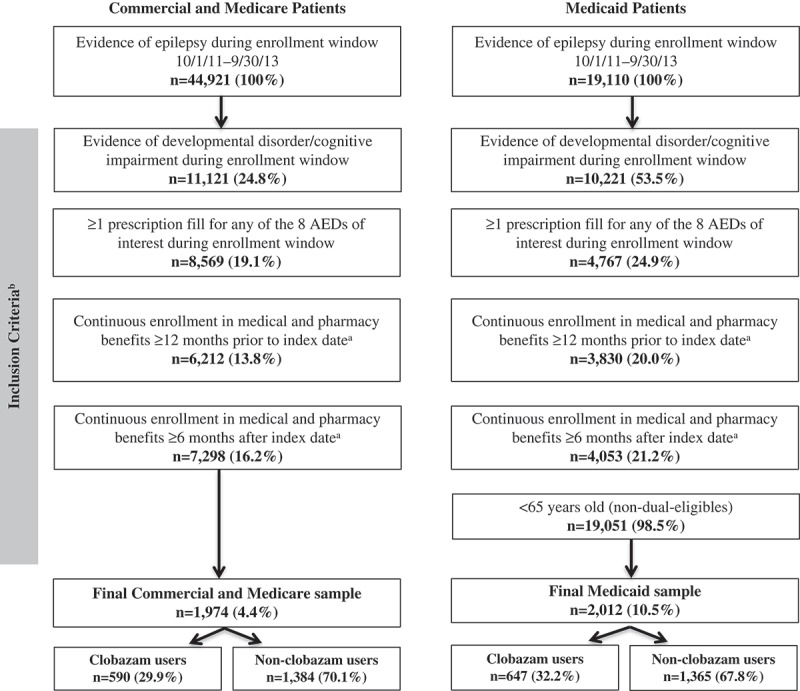
Non-mutually exclusive sample attrition commercial and medicare patients. Notes: AED – antiepileptic drug. ^a^ Continuous enrollment could only be verified for patients with an AED prescription fill within the enrollment window, as only these patients were assigned an index date. ^b^ Not mutually exclusive.

A total of 44,921 Commercial/Medicare Supplemental patients and 19,110 Medicaid patients with evidence of epilepsy were identified during the enrollment window (Figure 1). After applying the study inclusion and exclusion criteria, the final Commercial/Medicare population consisted of 1974 LGS patients, with 590 (29.9%) clobazam users and 1384 (70.1%) non-clobazam users. The Medicaid population consisted of 2012 LGS patients, with 647 (32.2%) clobazam users and 1365 (67.8%) non-clobazam users. The average age of the Commercial/Medicare Supplemental population was 26.1 years (standard deviation [SD]: 21.9) and 50.9% were males (Table 2). Patients in the clobazam cohort were younger (14.8 ± 12.5 vs 31.0 ± 23.2, *P *< 0.001) and a higher proportion were males compared to the non-clobazam cohort (54.6% vs 49.3%, *P *= 0.034). The average age of the Medicaid population was 20.7 (±16.8) years old and 51.6% were males (Table 3). Clobazam users were again younger compared to non-clobazam users (13.7 ± 11.4 vs 24.1 ± 17.9, *P *< 0.001).

**Table 2.** Baseline demographic and clinical characteristics of commercial/Medicare Supplemental patients.

	Clobazam		Non-clobazam		
	N = 590		N = 1384		*P*-value^a^
Demographic characteristics					
Age in years, mean (SD)	14.8	(12.5)	31.0	(23.2)	**<0.001**
Age group in years, n (%)					
0–5	117	19.8%	116	8.4%	**<0.001**
6–12	221	37.5%	266	19.2%	
13–17	84	14.2%	165	11.9%	
18–34	120	20.3%	314	22.7%	
35–44	21	3.6%	117	8.5%	
45–54	15	2.5%	129	9.3%	
55–64	8	1.4%	131	9.5%	
65+	4	0.7%	146	10.5%	
Males, n (%)	322	54.6%	683	49.3%	**0.034**
Geographic region, n (%)					
Northeast	138	23.4%	309	22.3%	0.41
North central	139	23.6%	280	20.2%	
South	179	30.3%	461	33.3%	
West	118	20.0%	290	21.0%	
Unknown	16	2.7%	44	3.2%	
Urban, n (%)	529	89.7%	1224	88.4%	0.431
Plan type, n (%)					
FFS	11	1.9%	97	7.0%	**<0.001**
EPO/PPO	379	64.2%	891	64.4%	
HMO	73	12.4%	164	11.8%	
POS	49	8.3%	76	5.5%	
CDHP/HDHP	57	9.7%	112	8.1%	
Unknown	21	3.6%	44	3.2%	
Payer type, n (%)					
Commercial	585	99.2%	1223	88.4%	**<0.001**
Medicare	5	0.8%	161	11.6%	
					
Capitation, n (%)					
Yes	36	6.1%	98	7.1%	0.429
No	554	93.9%	1286	92.9%	
					
Index year, n (%)					
2011	2	0.3%	122	8.8%	**<0.001**
2012	421	71.4%	737	53.3%	
2013	167	28.3%	525	37.9%	
					
Clinical characteristics					
Baseline medication classes,^b^ n (%)					
AEDs and other seizure-related medications	565	95.8%	1067	77. 1%	**<0.001**
Benzodiazepines	364	61.7%	289	20.9%	**<0.001**
NMDA receptor blockers	304	51.5%	361	26.1%	**<0.001**
SVP2a binder	268	45.4%	595	43.0%	0.319
Valproic acid	240	40.7%	170	12.3%	**<0.0001**
Sodium channel inhibitor	192	32.5%	205	14.8%	**<0.001**
Sulfamate-substituted monosaccharide	139	23.6%	122	8.8%	**<0.001**
Zonisamide	105	17.8%	100	7.2%	**<0.001**
GABA analogues	51	8.6%	131	9.5%	0.564
T-type calcium channel	36	6.1%	58	4.2%	0.068
					
Number of unique prescription classes (non-AEDs), mean (SD)	5.8	(4.9)	6.6	(6.2)	**0.002**
Number of pre-index AED drugs of interest, mean (SD)	1.7	(1.1)	0.5	(0.7)	**<0.001**
Baseline comorbidities,^c^ n (%)					
Pain	221	37.5%	797	57.6%	**<0.001**
Any digestive/bowel disorder	230	39.0%	460	33.2%	**0.014**
Constipation	100	17.0%	143	10.3%	**<0.001**
Psychosis	142	24.1%	516	37.3%	**<0.001**
Migraine/headache	54	9.2%	377	27.2%	**<0.001**
Intellectual disorder	148	25.1%	156	11.3%	**<0.001**
Mild	13	2.2%	27	2.0%	0.716
Moderate	14	2.4%	26	1.9%	0.476
Severe/profound	58	9.8%	46	3.3%	**<0.001**
Other	101	17.1%	100	7.2%	**<0.001**
Anxiety	35	5.9%	289	20.9%	**<0.001**
Depression	25	4.2%	289	20.9%	**<0.001**
Behavioral disorder	114	19.3%	235	17.0%	0.212
Eating disorder	105	17.8%	80	5.8%	**<0.0001**
Sleep disorder	93	15.8%	238	17.2%	0.435
Walking/gait impairment	77	13.1%	160	11.6%	0.351
Cognitive disorder	42	7.1%	213	15.4%	**<0.001**
Stroke/TIA	9	1.5%	179	12.9%	**<0.001**
Arthritis	9	1.5%	157	11.3%	**<0.001**
ADHD	44	7.5%	96	6.9%	0.680
Obesity	7	1.2%	87	6.3%	**<0.001**
Coronary heart disease	3	0.5%	82	5.9%	**<0.001**

Notes: ADHD – attention deficit hyperactivity disorder; AED – antiepileptic drug; CDHP – consumer-driven health plan; EPO – exclusive provider organization; FFS – fee-for-service; GABA – gamma aminobutyric acid; GI – gastrointestinal; GP – general practitioner; HDHP – high-deductible health plan; HMO – health maintenance organization; NMDA – N-methyl-D-aspartate; POS – point of service; PPO – preferred provider organization; SD – standard deviation; SVP2a – synaptic vesicle protein 2A; TIA – transient ischemic attack.

^a^ *P *< 0.05. *P*-values were obtained using Chi-square tests for categorical variables and *t*-tests for continuous variables. Fisher’s exact test was used where Chi-square test was not valid due to cells having fewer than expected counts.

^b^ Less than 5% of patients in both cohorts received T-type calcium channel, GABA reuptake inhibitor, barbiturate, carbonic anhydrase inhibitor, and K-channel opener.

^c^ Less than 5% of patients in both cohorts had a diagnosis of cortical dysplasia, tuberous sclerosis, brain lesions, heart failure, and chronic renal disease.

Bold text indicates a statistically significant difference with a *p*-value < 0.05.

**Table 3.** Baseline demographic and clinical characteristics of Medicaid patients.

	Clobazam		Non-clobazam		
	N = 647		N = 1365		*P*-value^a^
Demographic characteristics					
Age in years, mean (SD)	13.7	(11.4)	24.1	(17.9)	**<0.001**
Age group in years, n (%)					
0–5	165	25.5%	208	15.2%	**<0.001**
6–12	206	31.8%	267	19.6%	
13–17	101	15.6%	157	11.5%	
18–34	134	20.7%	342	25.1%	
35–44	21	3.2%	141	10.3%	
45–54	12	1.9%	144	10.5%	
55–64	8	1.2%	106	7.8%	
Males, n (%)	331	51.2%	707	51.8%	0.790
Race, n (%)					
White	348	53.8%	691	50.6%	**<0.001**
Black	80	12.4%	307	22.5%	
Hispanic	35	5.4%	48	3.5%	
Other/unknown	184	28.4%	319	23.4%	
Capitation, n (%)					
Yes	183	28.3%	450	33.0%	**0.035**
No	464	71.7%	915	67.0%	
Reason for Medicaid eligibility, n (%)					
Blind/disabled individual	531	82.1%	960	70.3%	**<0.001**
Child (not of unemployed adult/not foster care)	73	11.3%	207	15.2%	
Other^b^	43	6.6%	198	14.5%	
Index year, n (%)					
2011	2	0.3%	133	9.7%	**<0.001**
2012	478	73.9%	846	62.0%	
2013	167	25.8%	386	28.3%	
Clinical characteristics					
Developmental and cognitive disorders, n (%)	597	92.3%	1130	82.8%	**<0.001**
Number of unique diagnoses, mean (SD)	3.0	(2.0)	2.0	(1.7)	**<0.001**
Baseline medication classes,^c^ n (%)					
AEDs and other seizure-related medications	633	97.8%	1116	81.8%	**<0.001**
Benzodiazepine	429	66.3%	404	29.6%	**<0.001**
NMDA receptor blocker	310	47.9%	384	28.1%	**<0.001**
SVP2a binder	306	47.3%	568	41.6%	**0.016**
Valproic acid	236	36.5%	209	15.3%	**<0.001**
Sodium channel inhibitor	217	33.5%	289	21.2%	**<0.001**
Sulfamate-substituted monosaccharide	176	27.2%	158	11.6%	**<0.001**
Zonisamide	146	22.6%	107	7.8%	**<0.001**
GABA analogue	49	7.6%	136	10.0%	0.083
Anxiolytics	233	36.0%	401	29.4%	**0.003**
Antipsychotics	93	14.4%	329	24.1%	**<0.001**
Hypnotics	116	17.9%	211	15.5%	0.161
Number of unique prescription classes (non-AEDs), mean (SD)	7.6	(5.9)	7.8	(6.9)	**0.444**
Number of pre-index AED drugs of interest, mean (SD)	1.7	(1.1)	0.5	(0.7)	**<0.001**
Baseline comorbidities,^d^ n (%)					
Pain	284	43.9%	813	59.6%	**<0.001**
Intellectual disorder	324	50.1%	481	35.2%	**<0.001**
Mild	61	9.4%	144	10.6%	0.437
Moderate	90	13.9%	138	10.1%	**0.012**
Severe/profound	135	20.9%	162	11.9%	**<0.001**
Other	214	33.1%	307	22.5%	**<0.001**
Any digestive/bowel disorder	301	46.5%	587	43.0%	0.138
Constipation	158	24.4%	244	17.9%	**0.001**
Psychosis	195	30.1%	617	45.2%	**<0.001**
Migraine/headache	71	11.0%	411	30.1%	**<0.001**
Depression	40	6.2%	377	27.6%	**<0.001**
Behavioral disorder	164	25.4%	374	27.4%	0.332
Anxiety	48	7.4%	318	23.3%	**<0.001**
Eating disorder	123	19.0%	104	7.6%	**<0.001**
Sleep disorder	106	16.4%	252	18.5%	0.255
Cognitive disorder	58	9.0%	211	15.5%	**<0.001**
ADHD	60	9.3%	173	12.7%	**0.026**
Walking/gait impairment	74	11.4%	150	11.0%	0.765
Obesity	29	4.5%	134	9.8%	**<0.001**
Arthritis	12	1.9%	132	9.7%	**<0.001**
Stroke/TIA	18	2.8%	96	7.0%	**<0.001**

Notes: ADHD – attention deficit hyperactivity disorder; AED – antiepileptic drug; GI – gastrointestinal; GP – general practitioner; NMDA – N-methyl-D-aspartate; SD – standard deviation; SVP2a – synaptic vesicle protein 2A; SD – standard deviation; TIA – transient ischemic attack.

^a^ *P *< 0.05. *P*-values were obtained using Chi-square tests for categorical variables and *t*-tests for continuous variables. Fisher’s exact test was used where Chi-square test was not valid due to cells having fewer than expected counts.

^b^ Other includes aged individual, adult (not based on unemployed status), foster care child, and eligibility status unknown.

^c^ Less than 5% of patients in both cohorts received T-type calcium channel, GABA reuptake inhibitor, barbiturate, carbonic anhydrase inhibitor, and K-channel opener.

^d^ Less than 5% of patients in both cohorts had a diagnosis of cortical dysplasia, tuberous sclerosis, brain lesions, heart failure, chronic renal disease, and coronary heart disease.

Bold text indicates a statistically significant difference with a *p*-value < 0.05.

### Baseline clinical characteristics

Among the Commercial/Medicare Supplemental patients, compared to the non-clobazam cohort, higher proportions of the clobazam cohort had pre-index prescription fills for AEDs and other seizure-related medications (95.8% vs 77.1%, *P *< 0.001) and anxiolytics (35.1% vs 28.8%, *P *= 0.006), and a lower proportion had pre-index prescription fills for antipsychotics (10.3% vs 14.8%, *P *= 0.008) (Table 2). The clobazam cohort also had evidence of using a higher number of unique AED agents (1.7 ± 1.1 vs 0.5 ± 0.7, *P *< 0.001) and a lower number of non-AED prescription classes (5.8 ± 4.9 vs 6.6 ± 6.2, *P *= 0.002) prior to index. Significant differences were observed in the majority of comorbidities assessed, with the prevalence being lower in the clobazam cohort than in the non-clobazam cohort, with the following exceptions: tuberous sclerosis (2.7% vs 0.3%; *P *< 0.001), cortical dysplasia (3.1% vs 1.2%, *P *= 0.005), intellectual disorder (25.1% vs 11.3%, *P *< 0.001), eating disorder (17.8% vs 5.8%, *P *< 0.001), and digestive/bowel disorder (39.0% vs 33.2%, *P *= 0.014).

Among LGS Medicaid patients, baseline use of AEDs, other seizure-related medications, and non-AEDs was similar to that observed in the Commercial/Medicare Supplemental patients, with the exception that the clobazam cohort had claims for a similar number of pre-index non-AED prescription classes as the non-clobazam cohort. Baseline comorbidity differences were similar to the Commercial/Medicare Supplemental population with one exception: there was no significant difference in the baseline rates of digestive/bowel disorder between the cohorts.

### Baseline seizure-related resource utilization and costs

Commercial/Medicare Supplemental clobazam users had higher pre-index seizure-related resource utilization and associated costs compared to non-clobazam users (Table 4). Higher proportions of clobazam users had at least one seizure-related hospitalization (36.8% vs 27.5%, *P *< 0.001), physician office visit (94.6% vs 78.1%, *P *< 0.001), neurologist visit (58.8% vs 52.7%, *P *= 0.012), laboratory visit (43.1% vs 19.8%, *P *< 0.001), and other outpatient visit (67.5% vs 46.2%, *P *< 0.001). Clobazam users also had higher average seizure-related total inpatient length of stay (LOS) (2.6 ± 7.6 days vs 1.4 ± 4.7 days, *P *= 0.001) and higher average numbers of seizure-related visits by setting of care than non-clobazam users.

**Table 4.** Baseline healthcare utilization of commercial/Medicare Supplemental patients and Medicaid patients.

	Commercial/Medicare Supplemental					Medicaid^b^				
	Clobazam		Non-clobazam			Clobazam		Non-clobazam		
	N = 590		N = 1384		*P*-value^a^	N = 647		N = 1365		*P*-value^a^
Seizure-related health care utilization										
Hospitalization, n (%)	217	36.8%	381	27.5%	**<0.001**	209	32.3%	296	21.7%	**<0.001**
Number of inpatient visits, mean (SD)	0.7	(1.3)	0.4	(0.8)	**<0.001**	0.5	(1.1)	0.3	(0.8)	**<0.001**
LOS, mean (SD)	2.6	(7.6)	1.4	(4.7)	**0.001**	2.2	(6.7)	1.4	(6.0)	**0.010**
ER visits, n (%)	177	30.0%	418	30.2%	0.929	232	35.9%	539	39.5%	0.118
Number of ER visits, mean (SD)	0.6	(1.3)	0.5	(1.0)	0.216	0.8	(2.0)	0.9	(1.8)	0.365
Physician office visits, n (%)	558	94.6%	1081	78.1%	**<0.001**	577	89.2%	1033	75.7%	**<0.001**
Number of physician office visits, mean (SD)	3.9	(3.5)	2.2	(2.3)	**<0.001**	3.5	(3.2)	2.1	(2.2)	**<0.001**
Neurologist visit, n (%)	347	58.8%	729	52.7%	**0.012**	60	9.3%	99	7.3%	0.117
Number of neurologist visits, mean (SD)	1.8	(2.2)	1.3	(1.7)	**<0.001**	0.3	(1.1)	0.2	(0.8)	**0.024**
PCP visit, n (%)	115	19.5%	236	17.1%	0.194	177	27.4%	390	28.6%	0.572
Number of PCP visits, mean (SD)	0.5	(1.6)	0.3	(1.0)	**0.010**	0.8	(1.8)	0.7	(1.5)	0.304
Laboratory visits, n (%)	254	43.1%	274	19.8%	**<0.001**	163	25.2%	225	16.5%	**<0.001**
Number of laboratory visits, mean (SD)	1.1	(2.2)	0.3	(0.9)	**<0.001**	0.5	(1.2)	0.3	(0.9)	**<0.001**
Radiology visits, n (%)	58	9.8%	110	8.0%	0.170	55	8.5%	113	8.3%	0.866
Number of radiology visits, mean (SD)	0.1	(0.4)	0.1	(0.3)	0.052	0.1	(0.4)	0.1	(0.4)	0.830
Other outpatient visits, n (%)	398	67.5%	640	46.2%	**<0.001**	391	60.4%	588	43.1%	**<0.001**
Number of other outpatient visits, mean (SD)	7.1	(23.2)	1.8	(11.5)	**<0.001**	10.5	(38.3)	4.7	(27.6)	**0.001**
**All-cause healthcare utilization**										
Hospitalization, n (%)	303	51.4%	708	51.2%	0.935	309	47.8%	614	45.0%	0.243
Number of inpatient visits, mean (SD)	1.1	(1.7)	1.2	(1.8)	0.670	1.0	(1.8)	1.1	(2.0)	0.162
LOS, mean (SD)	5.2	(12.5)	6.5	(18.2)	0.081	5.0	(15.3)	6.7	(19.8)	**0.030**
ER visits, n (%)	333	56.4%	966	69.8%	**<0.001**	420	64.9%	998	73.1%	**<0.001**
Number of ER visits, mean (SD)	1.6	(2.4)	2.1	(3.2)	**<0.001**	2.3	(3.4)	3.4	(5.2)	**<0.001**
Physician office visits, n (%)	588	99.7%	1361	98.3%	**0.016**	636	98.3%	1310	96.0%	**0.006**
Number of physician office visits, mean (SD)	10.9	(7.4)	10.2	(7.6)	0.061	10.7	(7.9)	8.9	(7.2)	**<0.001**
Neurologist visit,^b^ n (%)	378	64.1%	878	63.4%	0.791	67	10.4%	135	9.9%	0.746
Number of neurologist visits, mean (SD)	2.2	(2.5)	1.9	(2.3)	**0.010**	0.4	(1.5)	0.3	(1.1)	0.082
PCP visit,^b^ n (%)	268	45.4%	852	61.6%	**<0.001**	321	49.6%	712	52.2%	0.286
Number of PCP visits, mean (SD)	2.1	(4.0)	3.2	(4.6)	**<0.001**	3.1	(5.1)	3.4	(5.2)	0.253
Laboratory visits, n (%)	406	68.8%	871	62.9%	**0.012**	379	58.6%	747	54.7%	0.104
Number of laboratory visits, mean (SD)	2.3	(3.0)	1.9	(3.0)	**0.002**	1.7	(3.9)	1.8	(3.5)	0.726
Radiology visits, n (%)	255	43.2%	768	55.5%	**<0.001**	254	39.3%	621	45.5%	**0.008**
Number of radiology visits, mean (SD)	0.9	(1.5)	1.4	(2.9)	**<0.001**	0.7	(1.2)	1.0	(1.7)	**<0.001**
Other outpatient visits, n (%)	567	96.1%	1329	96.0%	0.937	640	98.9%	1335	97.8%	0.082
Number of other outpatient visits, mean (SD)	33.0	(52.3)	18.1	(31.3)	**<0.001**	90.9	(91.4)	63.9	(87.7)	**<0.001**

Notes: ER – emergency room; LOS – length of stay; PCP – primary care physician; SD – standard deviation; USD – United States dollars.

^a^ *P *< 0.05. *P*-values were obtained using Chi-square tests for categorical variables and *t*-tests for continuous variables.

^b^ The majority of claims do not have information on physician specialty.

Bold text indicates a statistically significant difference with a *p*-value < 0.05.

The higher seizure-related resource utilization among the clobazam cohort led to higher average annual seizure-related costs over the pre-index period. Average annual pre-index seizure-related total ($33,478 ± $58,431 vs $12,709 ± $36,420, *P *< 0.001), medical ($24,066 ± $56,142 vs $10,563 ± $35,194, *P *< 0.001), and prescription ($9411 ± $12,416 vs $2146 ± $5596, *P *< 0.001) costs were significantly higher for clobazam users compared to non-clobazam users. Pre-index average medical costs by setting of care among patients in the clobazam cohort were two to four times greater than those of the non-clobazam cohort.

Medicaid clobazam users also had higher seizure-related resource utilization, on average, over the 12-month pre-index period compared to non-clobazam users (Table 4), with two exceptions. Unlike the Commercial/Medicare Supplemental patients, no significant differences were observed between the proportion of patients with a seizure-related neurologist visit or in the number of primary care provider (PCP) visits. Overall, pre-index cost differences were similar to the Commercial/Medicare Supplemental population, except there were no significant differences between the clobazam and non-clobazam cohorts in the cost of seizure-related neurologist visits.

### Baseline all-cause resource utilization and costs

Commercial/Medicare Supplemental clobazam users had higher all-cause resource utilization and associated costs, on average, over the 12-month pre-index period compared to non-clobazam users (Tables 4 and 5). Higher proportions of clobazam users had at least one physician office visit (99.7% vs 98.3%, *P *= 0.016) and laboratory visit (68.8% vs 62.9%, *P *= 0.012) any time during the pre-index period compared to non-clobazam users. However, the proportion of patients with at least one all-cause ER visit (56.4% vs 69.8%, *P *< 0.001) and radiology visit (43.2% vs 55.5%, *P *< 0.001) was lower among clobazam users than non-clobazam users. The average number of neurologist visits (2.2 ± 2.5 vs 1.9 ± 2.3, *P *= 0.010), laboratory visits (2.3 ± 3.0 vs 1.9 ± 3.0, *P *= 0.002), and other outpatient visits (33.0 ± 52.3 vs 18.1 ± 31.3, *P *< 0.001) was higher for clobazam users than non-clobazam users. Average annual pre-index all-cause total ($73,486 ± $110,918 vs $49,632 ± $89,843, *P *< 0.001), medical ($58,116 ± $100,102 vs $43,866 ± $86,376, *P *< 0.001), and prescription ($15,370 ± $36,617 vs $5766 ± $18,940, *P *< 0.001) costs were significantly higher for clobazam users compared to non-clobazam users.

**Table 5.** Baseline healthcare costs of commercial/Medicare Supplemental patients and Medicaid patients.

	Commercial/Medicare Supplemental					Medicaid^b^				
	Clobazam		Non-clobazam			Clobazam		Non-clobazam		
	N = 590		N = 1384		*P*-value^a^	N = 647		N = 1365		*P*-value^a^
Annual seizure-related costs in 2014 USD, mean (SD)										
Total	$33,478	(58,431)	$12,709	(36,420)	**<0.001**	$23,219	(84,306)	$7687	(25,092)	**<0.001**
Medical	$24,066	(56,142)	$10,563	(35,194)	**<0.001**	$15,544	(82,673)	$5951	(24,374)	**<0.001**
Hospitalization	$15,013	(48,020)	$7366	(30,951)	**<0.001**	$10,016	(80,795)	$4108	(22,651)	**<0.001**
ER	$961	(3549)	$793	(2314)	0.933	$297	(941)	$353	(979)	0.164
Physician office	$1581	(3770)	$562	(1438)	**<0.001**	$654	(1675)	$246	(699)	**<0.001**
Neurologist	$384	(577)	$214	(363)	**<0.001**	$33	(149)	$15	(80)	0.085
PCP	$115	(382)	$51	(190)	0.077	$66	(183)	$53	(247)	0.355
Laboratory	$350	(1434)	$82	(621)	**<0.001**	$28	(172)	$16	(98)	**<0.001**
Radiology	$146	(805)	$126	(632)	0.138	$57	(375)	$43	(212)	0.593
Other outpatient	$6015	(22,108)	$1634	(11,555)	**<0.001**	$4491	(16,426)	$1186	(8086)	**<0.001**
Prescription	$9411	(12,416)	$2146	(5596)	**<0.001**	$7676	(12,866)	$1736	(4262)	**<0.001**
Annual all-cause costs in 2014 USD, mean (SD)										
Total	$73,486	(110,918)	$49,632	(89,843)	**<0.001**	$62,989	(120,325)	$38,370	(70,243)	**<0.001**
Medical	$58,116	(100,102)	$43,866	(86,376)	**<0.001**	$49,667	(105,654)	$34,292	(68,737)	**<0.001**
Hospitalization	$30,441	(81,555)	$24,727	(70,535)	0.168	$17,858	(96,153)	$14,498	(58,921)	0.076
ER	$3450	(8543)	$4671	(9646)	**<0.001**	$1404	(3194)	$2036	(4009)	**<0.001**
Physician office	$4502	(7071)	$2841	(5036)	**<0.001**	$2516	(3508)	$1579	(2738)	**<0.001**
Neurologist	$483	(681)	$344	(524)	**<0.001**	$52	(262)	$27	(151)	0.576
PCP	$419	(1124)	$476	(1275)	**<0.001**	$254	(791)	$239	(538)	0.874
Laboratory	$679	(1978)	$369	(1163)	**<0.001**	$103	(278)	$101	(253)	**0.038**
Radiology	$542	(1799)	$930	(3911)	**<0.001**	$196	(639)	$224	(586)	**0.010**
Other outpatient	$18,500	(40,352)	$10,329	(27,363)	**<0.001**	$27,590	(39,437)	$15,854	(28,874)	**<0.001**
Prescription	$15,370	(36,617)	$5766	(18,940)	**<0.001**	$13,321	(45,428)	$4078	(7315)	**<0.001**

Notes: ER – emergency room; LOS – length of stay; PCP – primary care physician; SD – standard deviation; USD – United States dollars.

^a^ *P *< 0.05. *P*-values for differences in cost were obtained using the Wilcoxon-Mann-Whitney test.

^b^ The majority of claims do not have information on physician specialty.

Bold text indicates a statistically significant difference with a *p*-value < 0.05.

Overall, Medicaid clobazam users also had higher all-cause resource utilization and total all-cause costs, on average, over the 12-month pre-index period compared to non-clobazam users (Tables 4 and 5).

### Treatment patterns: clobazam and non-clobazam users

Clinically and statistically significant differences were observed between the baseline demographic and clinical profiles of clobazam and non-clobazam users. Therefore, adjustments to ameliorate selection bias prior to comparison of outcomes were required. Assessment of the standardized mean differences after applying the standardized mortality ratio weighting showed that many of the baseline characteristics remained unbalanced between the cohorts. Additional assessments were performed including limiting the study population and matching on few variables before the standardized mortality ratio weighting; however, regardless of method, the baseline characteristics between the clobazam and non-clobazam cohorts remained unbalanced (results not shown). Therefore, no meaningful statistical inference can be drawn on the comparison of the clobazam and non-clobazam users, so only univariate analyses were performed and reported for the assessment of treatment patterns.

Among the Commercial/Medicare Supplemental population, over the six months post-index, clobazam users had, on average, 5.5 (±2.6) clobazam prescription fills and stayed on treatment for an average of 140 (±50.8) days and on all AED treatment for an average of 166.0 (±29.6) days (Table 6). Non-clobazam users had, on average, 4.6 (±2.8) index prescription fills and stayed on index treatment for an average of 119.5 (±62.6) days and on all AED treatment for an average of 138.4 (±54.0) days over the same post-index period. Assessment of changes to the index therapy showed that within the clobazam cohort, 11.4% augmented, 27.5% discontinued, and 56.3% continued clobazam therapy. Among the non-clobazam users, 9.2% augmented, 38.4% discontinued, and 45.9% continued their index therapy. Approximately 11.5% of clobazam users and 33.1% of non-clobazam users discontinued all AED therapy during the six-month post-index period. Results over 12-months post-index treatment were consistent with the six-month results.

**Table 6.** Treatment patterns of commercial/Medicare Supplemental patients and Medicaid patients.

	Commercial/Medicare Supplemental				Medicaid			
	Clobazam		Non-clobazam		Clobazam		Non-clobazam	
Treatment Patterns	N = 590		N = 1384		N = 647		N = 1365	
Number of index AED prescription claims, mean (SD)	5.5	(2.6)	4.6	(2.8)	5.7	(2.6)	4.6	(2.7)
Total days on index AED, mean (SD)	140.1	(50.8)	119.5	(62.6)	138.7	(51.5)	114.1	(64.3)
Changes to index treatment, n (%)								
Augmentation	67	11.4%	127	9.2%	65	10.1%	111	8.1%
Switching	29	4.9%	91	6.6%	30	4.6%	86	6.3%
Discontinuation	162	27.5%	531	38.4%	162	25.0%	545	39.9%
Continued index therapy	332	56.3%	635	45.9%	390	60.3%	623	45.6%
Time to change in index treatment in days, mean (SD)								
Time to augmentation	60.8	(50.2)	49.6	(55.6)	70.6	(58.0)	59.3	(63.1)
Time to switch	76.2	(50.9)	69.0	(51.0)	99.4	(47.0)	59.0	(46.1)
Time to discontinuation	60.4	(36.8)	47.9	(35.4)	55.0	(33.6)	45.1	(36.9)
								
Total days on any AED,^a^ mean (SD)	166.0	(29.6)	138.4	(54.0)	164.4	(33.3)	134.5	(56.8)
All AED^a^ discontinuation rate, n (%)	68	11.5%	458	33.1%	72	11.1%	462	33.8%
Time to discontinuation from all AEDs^a^ in days, mean (SD)	71.4	(37.9)	52.3	(36.6)	62.4	(35.0)	49.5	(38.3)

Notes: AED – antiepileptic drug; SD – standard deviation.

^a^ Eight AEDs were considered in the study.

Among Medicaid patients, clobazam users had, on average, 5.7 (±2.6) index prescription fills and stayed on clobazam for an average of 138.7 (±51.5) days and on any AED for an average of 164.4 (±33.3) days over the six months of the post-index period (Table 6). Non-clobazam users had, on average, 4.6 (±2.7) index prescription fills and stayed on their index AED for an average of 114.1 (±64.3) days and on any AED for an average of 134.5 (±56.8) days over the six months of the post-index period. Assessment of changes to the index AED showed that among clobazam users, 10.1% augmented, 25.0% discontinued, and 60.3% continued clobazam therapy. Among the non-clobazam users, 8.1% augmented, 39.9% discontinued, and 45.6% continued their index AED. Similar proportions of the clobazam and non-clobazam users in the Medicaid population discontinued all AED therapy; again, the 12-month results were consistent with the six-month results.

### Healthcare resource utilization and costs: clobazam: pre- vs post-clobazam treatment

**Figure 2.**
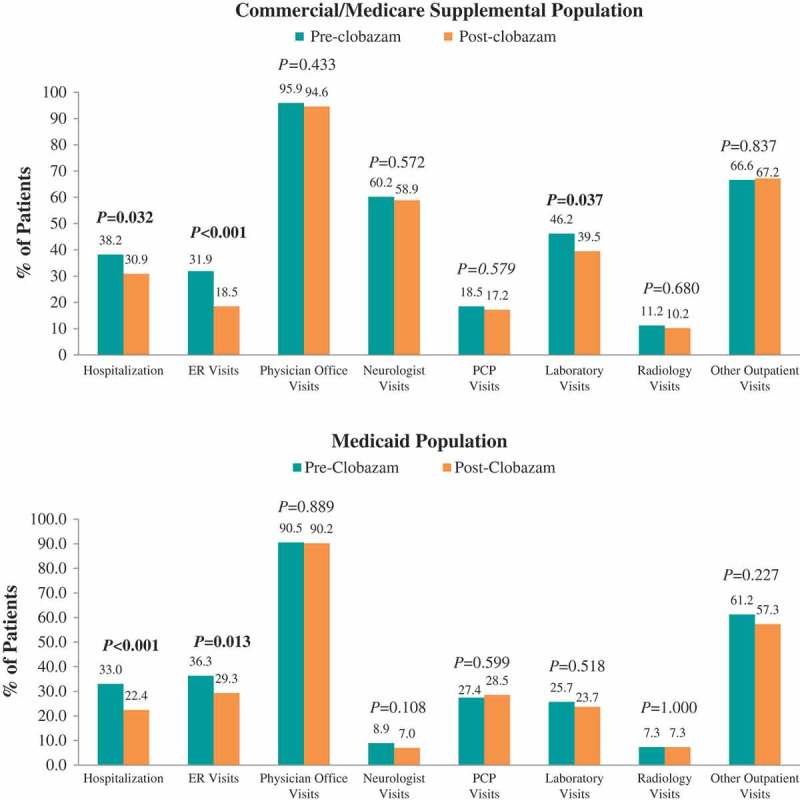
Proportion of patients utilizing seizure-related healthcare resources pre- vs post-clobazam. Notes: ER – emergency room; PCP – primary care physician. *P*-values in bold < 0.05. *P*-values for unadjusted differences in categorical variables were obtained using McNemar’s test to account for pre-post design.

**Figure 3.**
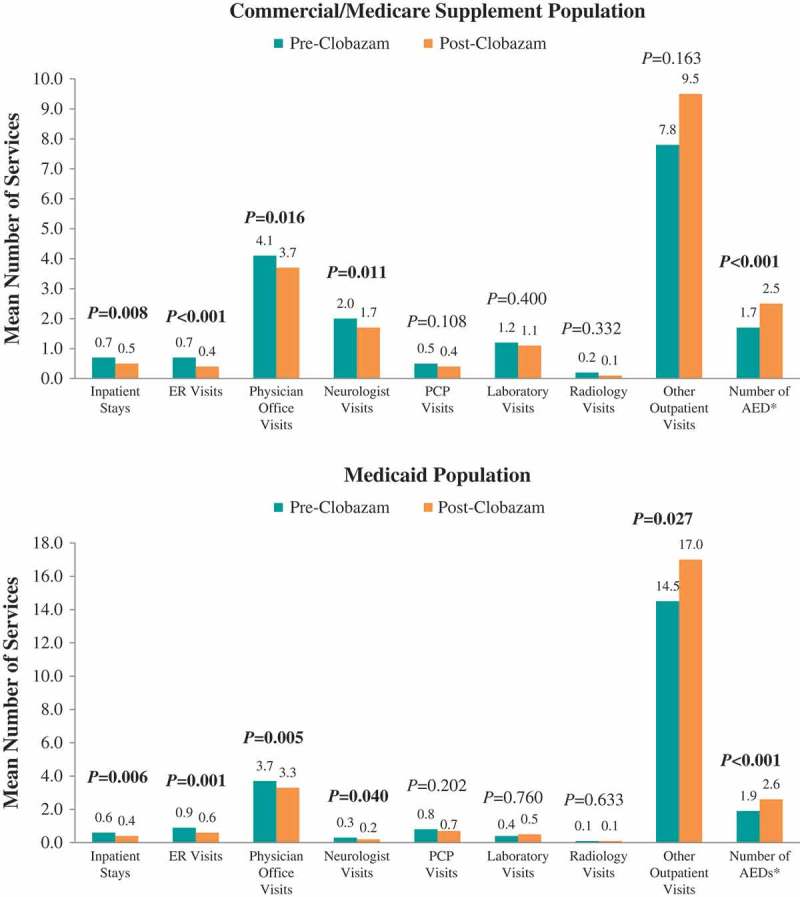
Mean number of seizure-related healthcare services utilized pre- vs post-clobazam. Notes: AED – antiepileptic drug; ER – emergency room; PCP – primary care physician. *P*-values in bold < 0.05. *P*-values for count variables were obtained using paired *t*-tests to account for pre-post design. *Includes clobazam.

**Figure 4.**
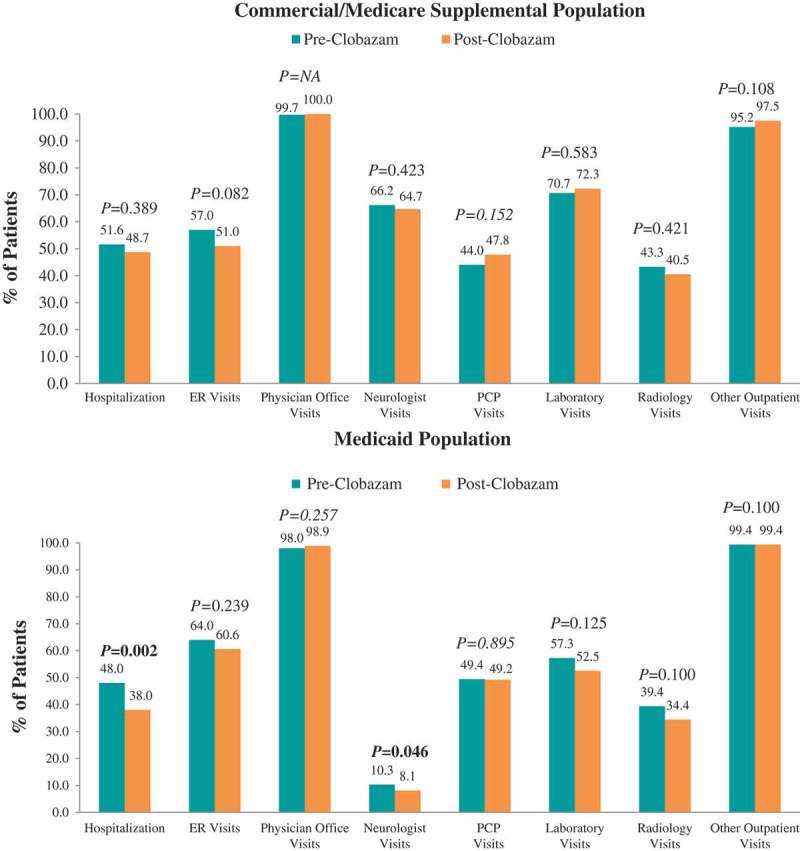
Proportion of patients utilizing all-cause healthcare resources pre- vs post-clobazam. Notes: ER – emergency room; PCP – primary care physician. *P*-values in bold < 0.05. *P*-values for unadjusted differences in categorical variables were obtained using McNemar’s test to account for pre-post design.

**Figure 5.**
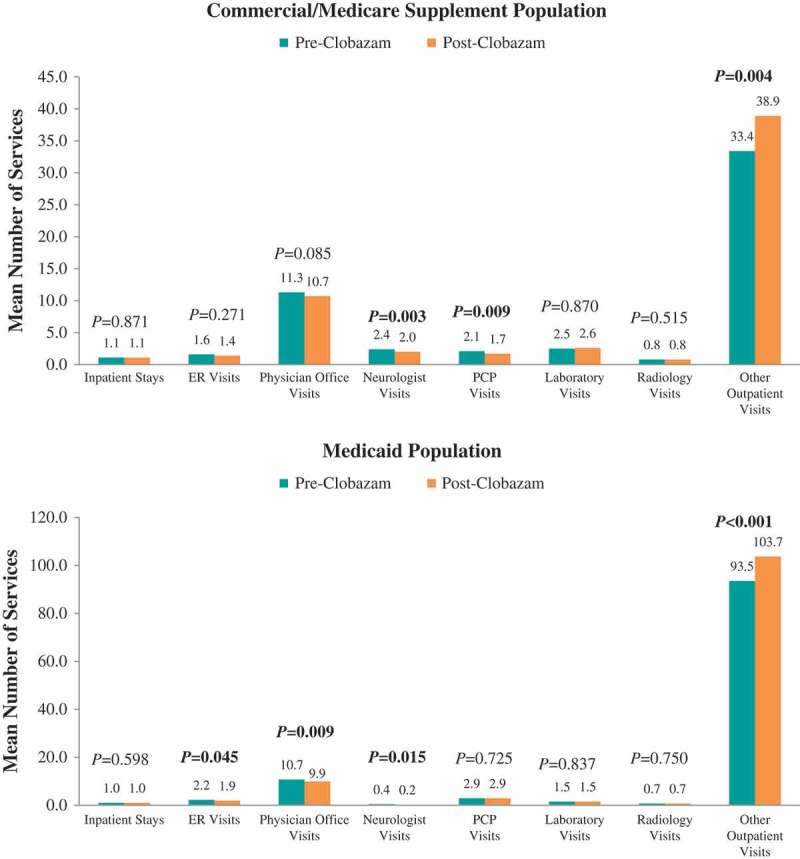
Mean number of all-cause healthcare services utilized pre- vs post-clobazam. Notes: ER – emergency room; PCP – primary care physician. *P*-values in bold < 0.05. *P*-values for count variables were obtained using paired *t*-tests to account for pre-post design.

Of the 590 Commercial and Medicare Supplemental (647 Medicaid) clobazam users, 314 (358 Medicaid) were continuously enrolled in medical and pharmacy claims for at least 12 months post-index and had at least one clobazam prescription between 6 and 12 months post-index. Seizure-related and all-cause healthcare utilization data for the 12-month pre- and post-clobazam initiation periods are presented in Figures 2–5, while associated costs are presented in Table 7.

**Table 7.** Pre-index and post-index healthcare costs among clobazam patients in the commercial/Medicare Supplemental and Medicaid populations.

	Commercial/Medicare Supplemental (N = 314)					Medicaid (N = 358)				
	Pre-clobazam		Post-clobazam		*P*-value^a^	Pre-clobazam		Post-clobazam		*P*-value^a^
Healthcare costs										
Annual seizure-related costs in 2014 USD, mean (SD)										
Total	$33,289	(54,938)	$35,083	(46,149)	**0.029**	$27,541	(109,816)	$28,493	(44,016)	**<0.001**
Medical	$23,740	(52,288)	$19,958	(43,090)	**0.004**	$18,965	(108,221)	$14,442	(39,754)	**0.036**
Hospitalization	$13,711	(41,266)	$11,639	(32,128)	0.094	$12,608	(106,674)	$7805	(35,724)	**0.006**
ER	$908	(2612)	$662	(3441)	**0.002**	$304	(892)	$196	(502)	**0.003**
Physician office	$1873	(4673)	$1278	(1981)	**0.005**	$728	(1422)	$657	(1485)	**0.001**
Neurologist	$448	(672)	$355	(508)	**0.022**	$30	(142)	$17	(77)	0.100
PCP	$116	(391)	$81	(298)	0.072	$76	(212)	$65	(194)	0.303
Laboratory	$397	(1385)	$270	(1026)	0.177	$34	(226)	$46	(324)	0.378
Radiology	$124	(689)	$92	(476)	0.366	$58	(436)	$29	(181)	0.336
Other outpatient	$6727	(25,573)	$6018	(20,393)	0.733	$5234	(17,472)	$5708	(17,619)	0.263
Prescription	$9549	(12,578)	$15,125	(13,735)	**<0.001**	$8576	(14,445)	$14,051	(16,375)	**<0.001**
Annual all-cause costs in 2014 USD, mean (SD)										
Total	$73,319	(117,534)	$81,389	(110,776)	**<0.001**	$68,565	(150,749)	$73,153	(102,307)	**<0.001**
Medical	$57,090	(107,206)	$59,292	(93,302)	0.411	$52,667	(131,965)	$52,937	(89,727)	0.880
Hospitalization	$28,079	(84,526)	$29,097	(57,815)	0.308	$20,860	(124,848)	$18,809	(70,923)	0.506
ER	$3228	(7792)	$3128	(9189)	0.102	$1382	(2714)	$1194	(2122)	0.421
Physician office	$4984	(8060)	$4243	(5961)	0.076	$2596	(3365)	$2431	(3329)	**0.024**
Neurologist	$546	(763)	$457	(686)	**0.013**	$50	(290)	$23	(113)	0.055
PCP	$456	(1317)	$311	(777)	0.060	$235	(447)	$236	(423)	0.509
Laboratory	$725	(1965)	$580	(1525)	0.294	$111	(333)	$130	(516)	0.309
Radiology	$471	(1467)	$341	(978)	0.214	$203	(715)	$131	(404)	**0.028**
Other outpatient	$19,603	(47,316)	$21,903	(48,312)	0.073	$27,516	(37,829)	$30,242	(40,282)	0.092
Prescription	$16,229	(35,832)	$22,098	(44,556)	**<0.001**	$15,898	(58,577)	$20,215	(36,680)	**<0.001**

Notes: ER – emergency room; PCP – primary care physician; SD – standard deviation; USD – United States dollars.

^a^ *P*-values in bold <0.05. *P*-values for unadjusted differences in categorical variables were obtained using McNemar’s test to account for pre-post design. Similarly, *P*-values for count variables were obtained using paired *t*-tests. *P*-values for difference in mean costs were obtained using Wilcoxon signed-rank test (non-parametric version of a paired *t*-test) and *P*-values for difference in median costs were obtained using Wilcoxon signed rank test.

Bold text indicates a statistically significant difference with a *p*-value < 0.05.

#### Seizure-related resource utilization and costs

The proportions of patients hospitalized (38.2% vs 30.9%, *P *= 0.032) and receiving ER care (31.9% vs 18.5%, *P *< 0.001) post-clobazam initiation were lower compared with the period prior to clobazam initiation among the Commercial and Medicare Supplemental population (Figure 2). The mean numbers of seizure-related inpatient stays (0.7 ± 1.3 vs 0.5 ± 1.1, *P *= 0.008), ER visits (0.7 ± 1.4 vs 0.4 ± 1.2, *P *< 0.001), and neurologist visits (2.0 ± 2.3 vs 1.7 ± 2.0, *P *= 0.011) were also significantly lower post-clobazam initiation (Figure 3). Finally, the mean number of unique concomitant AED agents (excluding clobazam) decreased post-clobazam initiation (1.7 ± 1.1 vs 1.5 ± 1.0, *P *< 0.001).

Average annual seizure-related medical costs were significantly lower post-clobazam initiation ($23,740 ± $52,288 vs $19,958 ± $43,090, *P *= 0.004), driven by lower seizure-related ER ($908 ± $2612 vs $662 ± $3441, *P *= 0.002) and physician office visit ($1873 ± $4673 vs $1278 ± $1981, *P *= 0.005) costs (Table 7). However, there were significantly higher seizure-related prescription costs in the post-index period ($9549 ± $12,578 vs $15,125 ± $13,735, *P *< 0.001), which resulted in significantly higher average annual seizure-related total costs compared to before initiation ($33,289 ± $54,938 vs $35,083 ± $46,149, *P *= 0.029).

Among Medicaid patients, similar trends in seizure-related resource utilization and costs were observed over the 12 months post-clobazam initiation compared to pre-clobazam initiation. However, while the proportion of patients with lab visits did not differ, and average utilization for other outpatient services significantly increased post-clobazam initiation. Also in this population, the reduction in medical costs was driven predominantly by a reduction in seizure-related hospitalization costs.

#### All-cause resource utilization and costs

In the 12 months following clobazam initiation, there were no significant differences in the proportions of Commercial and Medicare Supplemental patients with all-cause resource utilization in specific settings compared with the 12 months prior to clobazam initiation (Figure 4). The mean number of neurologist visits (2.4 ± 2.5 vs 2.0 ± 2.2, *P *= 0.003) and PCP visits over 12 months significantly decreased (2.1 ± 4.1 vs 1.7 ± 3.2, *P *= 0.009) post-clobazam initiation compared with pre-clobazam initiation, while the number of other outpatient visits increased (33.4 ± 53.4 vs 38.9 ± 60.9, *P *= 0.004) (Figure 5).

Average annual all-cause medical costs were not significantly different post-clobazam initiation ($57,090 ± $107,206 vs $59,292 ± $93,302, *P *= 0.411) (Table 7). However, there were significantly higher all-cause prescription costs post-clobazam initiation ($16,229 ± $35,832 vs $22,098 ± $44,556, *P *< 0.001), resulting in significantly higher average annual all-cause total costs compared to pre-clobazam initiation ($73,319 ± $117,534 vs $81,389 ± $110,776, *P *< 0.001).

Among Medicaid patients, the proportion of patients with at least one hospitalization and neurologist visit significantly decreased from pre- to post-clobazam initiation. Post-clobazam initiation, the average number of ER visits, physician office visits, and neurologist visits decreased, while the number of other outpatient visits increased compared to pre-clobazam. The all-cause costs trend from the pre-clobazam period to the post-clobazam period was similar to that for the Commercial and Medicare Supplemental patients.

## Discussion

This study demonstrated that there are key differences in the demographic and clinical characteristics of probable LGS patients initiating treatment with clobazam compared to those initiating other AEDs in both the Commercial/Medicare Supplemental and Medicaid populations under study. Treatment patterns associated with the index AED and overall AED use also differed between these patients. In addition, among patients treated with clobazam, both seizure-related healthcare utilization and its associated medical costs declined when compared to an equivalent 12-month period prior to treatment initiation.

Patients who initiated treatment with clobazam were younger and received more seizure-related clinical care prior to treatment initiation than those who initiated treatment with alternative AEDs. Prior to treatment initiation, clobazam patients also had higher seizure-related healthcare utilization and incurred both higher all-cause and seizure-related costs compared to those treated with other AEDs. Results were presented for the overall population due to the similar trend observed when clobazam and non-clobazam patients were compared in pediatric and adult cohorts (analysis not presented).

Several statistical methods were employed in an attempt to create comparability between the baseline characteristics of the clobazam and non-clobazam cohorts; however, none were successful. These results highlight that LGS patients enrolled in this sample of employer-sponsored and Medicaid benefit plans and initiating clobazam treatment compared to other AED treatments during the same time period have greater disease severity, which suggests a physician prescribing preference for clobazam in LGS patients who have more severe epilepsy. Similar findings were observed in a database study conducted in adult epilepsy patients in the United Kingdom (UK). In that study, prior to treatment initiation, patients initiating clobazam treatment were younger and had greater use of concomitant AEDs than those receiving clonazepam [15].

Treatment pattern results were not statistically compared between the clobazam and non-clobazam cohorts due to the differences described; however, the unadjusted results indicate that patients treated with clobazam discontinued or switched treatment less often and stayed on treatment longer relative to patients initiating treatment with alternative AEDs. The lower rate of treatment discontinuation and switch among the clobazam users may be due to better treatment benefit with clobazam, resulting in patients remaining on treatment longer. Adverse events profile of these drugs might have also affected the rate of treatment discontinuation and switch. Treatment patterns in LGS patients have not been extensively evaluated; although in the UK study noted above, treatment duration differed from that reported in the current study [15]. The median treatment duration was similar between the epilepsy patients treated with clobazam or clonazepam. The observed differences could be a result of the duration of follow-up; the current study evaluated treatment patterns over both a 6- and 12-month period, while average follow-up in the UK study was at least five years.

Among patients treated with clobazam, healthcare resource utilization and its associated costs were compared among clobazam users at 12 months pre- and post-clobazam initiation. Results showed that all-cause and seizure-related resource utilization was significantly reduced post-clobazam initiation. Higher prescription costs post-clobazam initiation was mostly offset by the reduction in medical costs, due in part to a reduction in seizure-related hospitalizations and ER visits. These results are also consistent with previous findings that clobazam could have a positive overall economic impact through decreased seizure-related medical costs [11,12]. In addition to decreased seizure-related medical costs, clobazam may also have an impact on indirect costs, such as decreased caregiver time, resulting in less productivity loss, and decreased long-term disability due to head injury for children [16].

This study has inherent limitations due to the use of claims data. There is no specific ICD-9-CM diagnosis code for LGS, and while the patient identification algorithm used in this investigation was based on previously published criteria and clinical opinion, misclassification of LGS patients might still be present. However, our results still provide valuable insight into the use of AED and impact of clobazam on costs in LGS patients. Baseline characteristics of clobazam and non-clobazam users indicate that these are patients with different disease severity, and statistical methods employed to remove these differences were unsuccessful. As a result, differences in post-index measures could not be evaluated between the clobazam and non-clobazam cohorts. Furthermore, clinical data are unavailable in the database; therefore, clinical observations that might influence physicians to prescribe one AED vs another could not be evaluated, and instead proxy measures of baseline disease severity and comorbidity were used. Another limitation is the pre- and post-treatment design, which has a potential for regression to the mean effect. However, this bias is more likely to occur when there is a treatment switch during an acute episode, such as in schizophrenia [17], than in the case of LGS, a chronic condition in which clobazam is often added to existing treatments. Finally, since the analysis focused on burden specific to medical resource use, it provides no insight into the relative impact of clobazam and other AEDs with respect to clinically important outcomes such as patient quality of life, activities of daily living, and caregiver burden.

## Conclusions

Baseline comparisons suggest a prescribing preference for clobazam in LGS patients with more severe disease, regardless of whether the patient has healthcare benefits provided through private or public payers. The inability to eliminate selection bias further emphasizes the differences between the clobazam and non-clobazam treated cohorts. Regardless, clobazam users did have significant improvement in seizure-related resource utilization and medical costs post-clobazam initiation compared to the year prior to clobazam initiation.
